# Identification of New Prognostic Markers and Therapeutic Targets for Non-Muscle Invasive Bladder Cancer: HER2 as a Potential Target Antigen

**DOI:** 10.3389/fimmu.2022.903297

**Published:** 2022-05-23

**Authors:** Han Kyu Chae, Wook Nam, Han Gwun Kim, Sharon Lim, Byeong-Joo Noh, So Won Kim, Gil Hyun Kang, Jong Yeon Park, Dae-Woon Eom, Sung Jin Kim

**Affiliations:** ^1^ Department of Urology, Gangneung Asan Hospital, University of Ulsan College of Medicine, Gangneung, South Korea; ^2^ Department of Pathology, Gangneung Asan Hospital, University of Ulsan College of Medicine, Gangneung, South Korea; ^3^ Department of Parmacology, Asan Medical Center, University of Ulsan College of Medicine, Seoul, South Korea

**Keywords:** HER2 (human epidermal growth factor receptor-2), PD-L1, PD1, CD8, ki67, ADC (antibody-drug conjugate), BCG (Bacillus Calmette–Guérin)

## Abstract

Bacillus Calmette–Guérin (BCG) is the gold standard adjuvant treatment for non-muscle-invasive bladder cancer (NMIBC). However, given the current global shortage of BCG, new treatments are needed. We evaluated tumor microenvironment markers as potential BCG alternatives for NMIBC treatment. Programmed death-ligand 1, human epidermal growth factor receptor-2 (HER2), programmed cell death-1 (PD1), CD8, and Ki67 levels were measured in treatment-naïve NMIBC and MIBC patients (pTa, pT1, and pT2 stages). Univariate and multivariate Cox proportional hazard models were used to determine the impact of these markers and other clinicopathological factors on survival, recurrence, and progression. EP263, IM142, PD1, and Ki67 levels were the highest in the T2 stage, followed by the T1 and Ta stages. HER2 and IM263 expressions were higher in the T1 and T2 stages than in the Ta stage. In NMIBC, the significant prognostic factors for recurrence-free survival were adjuvant therapy, tumor grade, and HER2 positivity, whereas those for progression-free survival included age, T-stage, and IM263. Age, T-stage, EP263, PD1, CD8, and Ki67 levels were significant factors associated with overall survival. IM263 and HER2 are potential biomarkers for progression and recurrence, respectively. Therefore, we propose HER2 as a potential target antigen for intravesical therapeutics as a BCG alternative.

## Introduction

Urothelial carcinoma (UC) is the ninth most common cancer with the thirteenth highest mortality rate among cancers worldwide (1). Of all diagnosed bladder cancers, approximately 75% are non-muscle-invasive bladder cancer (NMIBC), and the remaining are muscle-invasive bladder cancer (MIBC) (2). High-risk NMIBC patients require adjuvant intravesical therapy due to the risk of recurrence and progression to MIBC.

Intravesical Bacillus Calmette–Guérin (BCG) for UC, the first immunotherapeutic anti-cancer agent approved by the US Food and Drug Administration (FDA) (3), still remains the gold standard for adjuvant treatment of high-risk NMIBC patients. BCG therapy is challenging in terms of its ineffectiveness and adverse effects. Even among patients who respond well to initial BCG treatment, 10-20% of patients relapse and eventually progress to MIBC (4, 5). Since no other effective treatments are available, additional use of BCG is recommended in such cases based on each patient’s situation, despite insufficient evidence of its effectiveness (6). Radical cystectomy is the only option for patients who fail to achieve remission after initial BCG therapy, which has high morbidity (2). Of the 15% of patients who discontinue treatment after first course of BCG treatment, 35% of them are known to have difficulty in continuing treatment due to side effects of bacterial or chemical cystitis, hematuria, and systemic febrile events (7). Globally, BCG shortage exists because the BCG strain (Connaught, Tice) is lacking (6). Research on reduction in dose and frequency of BCG treatment was also conducted; however, recurrence-related prognosis was reported to be inferior (8).Thus, developing an alternative therapy to BCG is necessary.

Recently, there have been several advancements in UC treatment, including immune checkpoint blockers (ICBs) (9, 10). Several clinical studies have evaluated the therapeutic efficacy of ICBs for high-risk NMIBC patients who have failed to respond to BCG therapy (11, 12). Enfortumab vedotin (EV), a novel antibody-drug conjugate (ADC) targeting nectin-4, was approved by the FDA as a therapeutic agent for metastatic UC (mUC) (13). Before the development of ADCs, human epidermal growth factor receptor-2 (HER2) received attention as a therapeutic target but was shown to be ineffective (14). Following the approval of monomethyl auristatin E (MMAE) as a cytotoxic agent in EV, RC48 has been used to develop a HER2-targeting ADC for the treatment of mUC (15). ADC’s novel mechanism is the direct injection of cytotoxic drugs into cancer cells. After identifying a target antigen, there is an opportunity to develop an intravesical therapeutic agent for this identified target antigen by mediating ADCs (16).

At a time when new trends are emerging in the field of anticancer drugs, we are interested in studying the underestimated potential of known biomarkers. Therefore, this study aimed to evaluate changes in the expression of tumor microenvironment markers, such as programmed death-ligand 1 (PD-L1), HER2, programmed death-1 (PD1), CD8, and Ki67, in NMIBC patients during the initial course of the disease and compare them with the expressions in MIBC patients to study the effects of tumor microenvironment and clinicopathological factors on recurrence, progression, and overall prognosis in NMIBC.

## Materials and Methods

This retrospective study was approved by the Institutional Review Board of Gangneung Asan Hospital, Gangneung, Republic of Korea (approval number: 2018-05-017) according to the principles of the Declaration of Helsinki. The requirement for informed consent was waived due to the retrospective nature of the study.

### Patients

**Figure 1:**
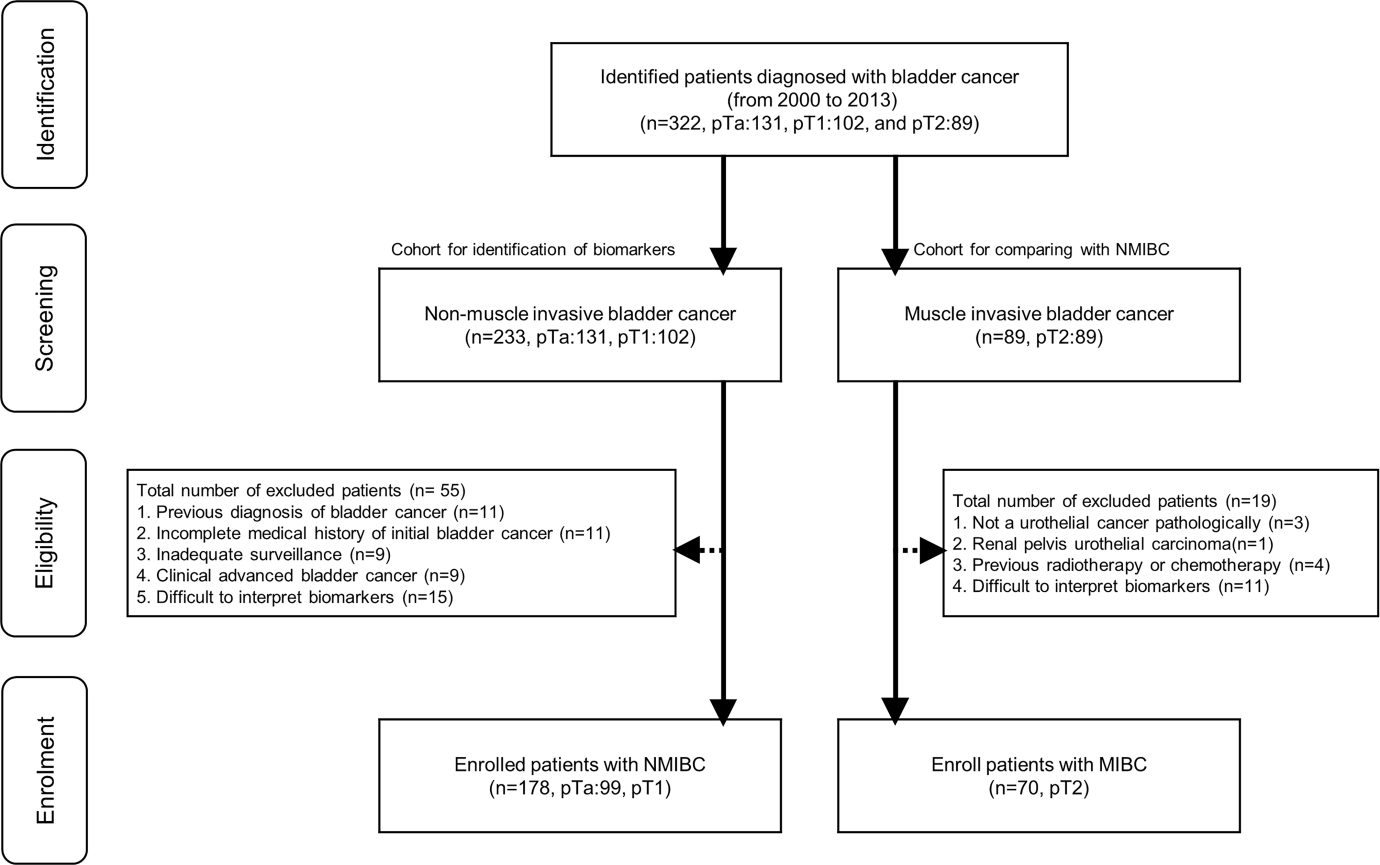
Flowchart for enrolled patients. NMIBC, non-muscle invasive bladder cancer; MIBC, muscle invasive bladder cancer.

Patients who had undergone transurethral resection of bladder tumor (TURB) between 2000 and 2013 (n=322, pTa: 131, pT1: 102, and pT2: 89) were screened for eligibility. We excluded 55 patients from 233 screened NMIBC patients (**Figure 1**). Exclusion criteria and the matched number of patients were as follows: 1) previous bladder cancer diagnosis (n=11); 2) incomplete medical history for initial bladder cancer (n=11); 3) inadequate follow-up for recurrence or progression (n=9); 4) clinically advanced bladder cancer that required invasive procedures (radical cystectomy, radiation therapy, and chemotherapy) (n=9); 5) pathological slides in which interpreting more than two microenvironmental markers after staining was difficult (n=15). Furthermore, we excluded 19 patients from 89 screened MIBC patients (**Figure 1**). Exclusion criteria and the matched number of patients were as follows: 1) pathologically, the bladder cancer was not a urothelial carcinoma (n=3); 2) renal pelvis urothelial carcinoma (n=1); 3) previous radiotherapy or chemotherapy (n=4); 5) pathological slides in which interpreting more than two microenvironmental markers after staining was difficult (n=11). We enrolled 248 patients with NMIBC (n=178, pTa: 99, pT1:79) and MIBC (n=70, pT2:70). All included NMIBC patients were diagnosed with bladder cancer for the first time at our hospital and had well-preserved specimens for analysis. The MIBC patients also had well-preserved pT2 specimens for analysis and clear medical records regardless of bladder cancer recurrence or adjuvant intravesical therapy. All patients were treatment-naïve and had no history of chemotherapy or radiotherapy. The expression levels of tumor microenvironment markers—EP263, EP142, IM263, IM142, HER2, PD1, CD8, and Ki67—were determined in all 248 patients. To evaluate prognosis related to survival, recurrence, and progression, we included clinicopathological factors (tumor number, tumor size, prior recurrence rate, T-stage, tumor grade, and association of carcinoma in situ) based on the European Organization for Research and Treatment of Cancer (EORTC) guidelines (17) in the analysis. Other patient information regarding sex, age, tumor characteristics, and history of adjuvant therapy were also collected.

### Follow-Up and Endpoint

The routine follow-up included cystoscopy performed every 3 months and upper tract imaging (computed tomography and ultrasonography) performed every year. Urine cytology was performed during cystoscopy. Lesions suspected of recurrence were biopsied during cystoscopy or elective surgery. Suspected lesions that were pathologically confirmed as UC were defined as recurrence, and the recurrence time was defined as the time when the suspected lesion was identified. Progression was defined as recurrent UC that was confirmed to be at a pathologically higher stage than the pT2 stage. Clinically definite progression was determined if the recurrent lesions required more invasive procedures (radical cystectomy, radiation therapy, and chemotherapy), and progression was also defined even if pT2 was not confirmed pathologically.

### Tissue Microarray

Formalin-fixed, paraffin-embedded tumor samples from specimens of 178 TURB diagnosed as UC were collected and arrayed using a tissue microarrayer (Quick-Ray^®^, Unitma Co., Ltd., Seoul, Republic of Korea). Briefly, representative areas from each tumor sample were selected and marked on hematoxylin and eosin-stained slides, and the corresponding tissue blocks were collected. Matching areas in each tumor block were punched with two tissue cylinders (2 mm diameter), and each core was transferred to its recipient microarray block. Sections (4 µm thick) were cut from the tissue microarray (TMA) paraffin blocks for immunohistochemical (IHC) staining.

### Immunohistochemistry

IHC staining of PD-L1 was performed using two different anti-PDL1 antibody clones (Ventana SP142 and SP263, Ventana Medical Systems, Tucson, USA) and the Benchmark automated staining system (Ventana) for the FDA-approved SP263 and SP142 assay kits, according to the package inserts. Rabbit monoclonal negative control immunoglobulin (Ventana) was used as a negative control. IHC staining of CD8 (SP16; Thermo Fisher Scientific, Runcorn, UK; 1:100) and PD1 (EPR4877; Abcam, Cambridge, UK; 1:100) was performed on TMA blocks using a Bond-Max automatic immunostaining device (Leica Biosystems, Newcastle, UK). IHC staining of HER2 (4B5; Roche Diagnostics, Tucson, USA; pre-dilution) and Ki67 (SP6; Cell Marque, California, USA; 1:300) was performed on TMA blocks using the Benchmark automated staining system (Ventana). As positive controls, we used placental tissue sections for PD-L1 and tonsil tissue sections for PD1, CD8, and Ki67. Negative controls were performed by omitting the primary antibody.

### Immunohistochemical Analyses

PD-L1 immunoreactivity was evaluated semi-quantitatively by a pathologist (DW EOM) blinded to the clinicopathological data. Membrane-based immunostaining of PD-L1 (SP263 and SP142) was interpreted based on the proportion of more than unequivocal positivity (any intensity) in both epithelial tumor cells and infiltrating immune cells. The proportion of positively stained epithelial tumor cells was graded as 0 (<1%), 1 (≥1% to <5%), 2 (≥5% to <50%), or 3 (≥50%). The proportion of positively stained tumor immune cells was graded as 0 (<1%), 1 (≥1% to <5%), 2 (≥5% to <10%), or 3 (≥10%). Based on the guideline’s interpretation, a positive 1%-cutoff value score was used (18).

**Figure 2:**
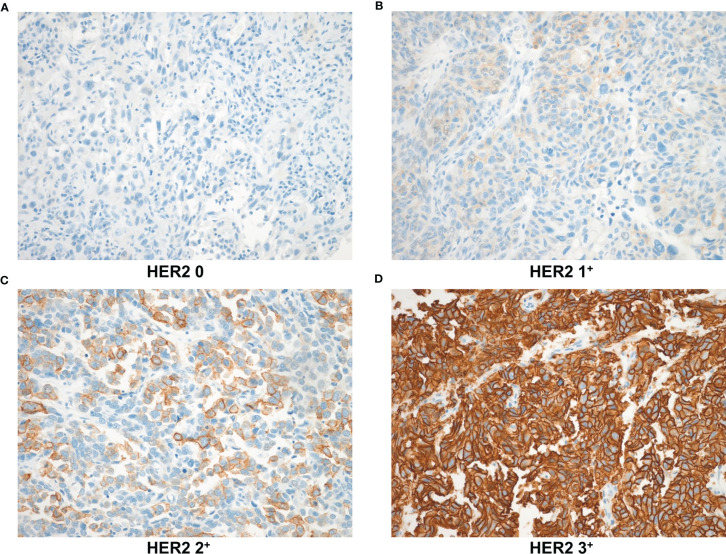
HER2 expression levels based on immunohistochemical staining were observed at 200× magnification. **(A)** HER2 0: no staining **(B)** HER2 1^+^: faint or partial membrane staining in ≤10% of tumor cells **(C)** HER2 2^+^: weak or moderate complete membrane staining in >10% of tumor cells **(D)** HER2 3^+^: strong complete membrane staining in >10% of tumor cells. HER2, human epidermal growth factor receptor-2.

Sections immunostained for PD1 and CD8 were assessed for tumor-infiltrating lymphocytes (TILs) in the tumor bed area. Immune cells were evaluated in each section by microscopic examination (400; BX51; Olympus, Tokyo, Japan). Five noncontiguous microscopic areas with TILs were randomly selected in each sample to corroborate representativeness and homogeneity. The mean number of immune cells in these five fields was calculated based on a 200× microscopic field (0.1590 mm^2^/field). The number of nuclear Ki67 positive cells (per 1,000 cells) was counted visually in the hotspots at high magnification, and the Ki67 value was scored by the labeling index (19). HER2 expression levels were examined according to breast cancer guidelines (20) as follows: HER2 0: no staining (**Figure 2A**, IHC score 0); HER2 1^+^: faint or partial membrane staining in ≤10% of tumor cells (**Figure 2B**, IHC score 1); HER2 2^+^: weak or moderate complete membrane staining in >10% of tumor cells (**Figure 2C**, IHC score 2); and HER2 3^+^: strong complete membrane staining in >10% of tumor cells (**Figure 2D**, IHC score 3). HER2+ (HER2 positive) staining was defined as expression levels of “HER2 1^+^ or HER2 2^+^ or HER2 3^+^” (**Figures 2B–D**).

### Statistical Analyses

We analyzed the relationship between bladder cancer-related outcomes and the variables previously mentioned using Pearson’s χ^2^ test or Fisher’s exact test. Continuous variables were analyzed using an independent t-test. The relationship between survival and clinicopathological factors was estimated using Kaplan–Meier models and analyzed using the log-rank test.

We also evaluated the impact of clinicopathological factors and microenvironmental markers on the overall survival (OS) in all stage (pTa-T2) patients using univariate and multivariate Cox proportional hazard models. To analyze recurrence-free survival (RFS) and progression-free survival (PFS) in NMIBC patients, variables were selected using the stepwise backward elimination method. All variables with p<0.2 in the univariate analysis were included in the multivariate analysis. Each multivariate model was built using PDL-1-related factors, including EP263, IM263, EP142, and IM142 along with the other variables, to prevent overlapping inclusion of factors. The multivariate model of NMIBC included factors that were found to be significant in the univariate analysis. Statistical significance was set at p<0.05. Statistical analyses were performed using the SPSS software (IBM Corp. Released 2011, version 20.0. Armonk, NY: IBM Corp.).

## Results

### Patient Clinicopathological Characteristics

**Table 1** summarizes the characteristics and clinical features of the patients according to disease stage (pTa, pT1, and pT2). We included 178 NMIBC patients in the analysis, 99 of which were diagnosed with pTa (39.9%), and 79 of which were diagnosed with pT1 (31.8%). To compare the tumor microenvironment between NMIBC and MIBC patients, we included 70 (28.2%) patients diagnosed with pT2 tumors (by TURB). Of the 248 patients, 201 (81.0%) were male. Patients in the pTa group tended to be younger than those in the pT1 or pT2 groups. Sex, diabetes, and hypertension were comparable among the three groups. Gross tumor appearance was evaluated; compared with pTa tumors, pT1 and pT2 tumors tended have a solid appearance more frequently (p<0.01). The proportion of patients with tumors >3 cm in size was higher in the pT1 group than in the pTa group and higher in the pT2 group than in the pT1 group (both p<0.01). The three groups showed no significant difference in tumor number. Pathological results were evaluated; regarding tumor grade, patients with pTa tumors had a lower incidence of high-grade disease at 40/93 (43.0%), whereas this incidence was 70/75 (93.3%) and 64/64 (100%) in patients with pT1 and pT2 tumors, respectively (p<0.01). The three groups showed no significant difference in the incidence of carcinoma in situ. Adjuvant therapy was administered to 40/99 (40.4%) patients in the pTa group and 44/79 (55.7%) in the pT1 group. Among the pTa patients, 27 (27.3%) received intravesical BCG, while 13 (13.1%) received intravesical mitomycin. Among the pT1 patients, 42 (53.2%) received intravesical BCG, and 2 (2.5%) received intravesical mitomycin. The median (interquartile range [IQR]) follow-up period in the pTa, pT1, and pT2 groups was 91.0 (69.5–122), 73.0 (38–123), and 16.0 (5–46.5) months, respectively. During the follow-up period, 58 (58.6%) pTa and 56 (70.9%) pT1 patients reported recurrence. The disease progressed to the pT2 stage in 2 (2.0%) pTa and 16 (20.3%) pT1 patients. During the follow-up period, 28 (28.3%), 42 (53.2%), and 62 (88.6%) patients expired in the pTa, pT1, and pT2 groups, respectively.

**Table 1:** Patient characteristics.

	Total	pTa	pT1	pT2	p-value
Number of patients, n (%)	248 (100)	99 (39.9)	79 (31.9)	70 (28.2)	
Age, years (mean ± SD)	69.3 ± 10.9	66.5 ± 11.0	71.3 ± 10.4	70.9 ± 10.7	<0.01^*^
Sex, n (%), Male	201 (81.0)	81 (81.8)	64 (81.0)	56 (80.0)	0.95^***^
BMI (kg/m^2^)	24.0 ± 2.8	24.8 ± 2.9	23.5 ± 2.6	23.2 ± 2.6	<0.01^**^
DM, n (%)	30 (12.1)	13 (13.1)	10 (12.7)	7 (10.0)	0.94^***^
HTN, n (%)	70 (28.2)	34 (34.3)	24 (30.4)	12 (17.1)	0.09^***^
Tumor description, n (%) Papillary Solid Mixed Not reported	247 (100) 191 (77.3) 11 (4.5) 45 (18.2) 1	99 (100) 96 (97.0) 0 (0.0) 3 (3.0) 0	79 (100) 64 (81.0) 6 (7.6) 9 (11.4) 0	69 (100) 31 (44.9) 5 (7.2) 33 (47.8) 1	<0.01^***^
Tumor grade, n (%) Low High Not reported	229 (100) 58 (25.3) 171 (74.7) 19	93 (100) 53 (57.0) 40 (43.0) 6	75 (100) 5 (6.7) 70 (93.3) 4	64 (100) 0 (0) 64 (100) 6	<0.01^***^
Tumor number, n (%) 1 2–4 >4 Not reported	219 (100) 151 (68.9) 57 (26.0) 11 (5.0) 29	88 (100) 56 (63.6) 27 (30.7) 5 (5.7) 11	68 (100) 43 (63.2) 20 (29.4) 5 (7.4) 11	63 (100) 52 (82.5) 10 (15.9) 1 (1.6) 7	0.09^***^
Tumor size (cm), n (%) 1 1–3 >3 Not reported	225 (100) 30 (13.3) 86 (38.2) 109 (48.5) 23	88 (100) 17 (19.3) 48 (54.5) 23 (26.1) 11	72 (100) 9 (12.5) 26 (36.1) 37 (51.4) 7	65 (100) 4 (6.2) 12 (18.5) 49 (75.4) 5	<0.01^***^
Concurrent CIS, n (%)	14 (5.6)	6 (6.1)	6 (7.6)	2 (2.9)	0.45^***^
Adjuvant therapy, n (%) BCG Mitomycin None	178 (100) 69 (38.8) 15 (8.4) 94 (52.8)	99 (100) 27 (27.3) 13 (13.1) 59 (59.6)	79 (100) 42 (53.2) 2 (2.5) 35 (44.3)	- - - -	
Recurrence, n (%)		58 (58.6)	56 (70.9)	–	0.12^***^
Progression, n (%)		2 (2.0)	16 (20.3)	–	<0.01^***^
Expire, n (%)	132 (53.2)	28 (28.3)	42 (53.2)	62 (88.6)	<0.01^***^

^*^Analysis of variance; ^**^Kruskal–Wallis; ^***^Pearson’s χ-2 test or Fisher’s exact test.

SD, standard deviation; BMI, body mass index; DM, diabetes mellitus; HTN, hypertension; CIS, carcinoma in situ; BCG, Bacillus Calmette–Guérin.

### Expression of Tumor Microenvironment Markers

**Figure 3:**
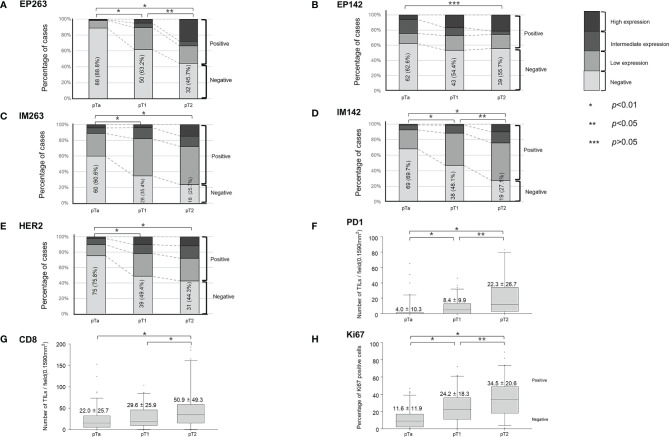
Baseline expression of tumor microenvironment markers according to T-stage. The percent of positive cases are shown for **(A)** EP263, **(B)** EP142, **(C)** IM263, **(D)** IM142, and **(E)** HER2 according to T-stage. The expression levels of **(F)** PD1, **(G)** CD8, and **(H)** Ki67 according to T-stage are also shown. TILs, tumor-infiltrating lymphocytes; HER2, human epidermal growth factor receptor-2; PD1, programmed cell death protein 1.

**Figure 3** and **Supplementary Table 1** show the expression of different markers of the tumor microenvironment at each stage of the disease. EP263 and IM142 positivity rates were significantly higher in the pT2 group than in the pTa and pT1 groups (**Figures 3A, D** and **Supplementary Table 1**). EP142 expression was significantly higher in pT2 patients than in pTa patients (**Figure 3B** and **Supplementary Table 1**). However, EP142 expression in pT1 patients was comparable to that in pTa and pT2 patients. The IM263 and HER2 positivity rates were higher in pT1 patients than in pTa patients, while no significant difference was noted between the pT1 and pT2 patients (**Figures 3C, E** and **Supplementary Table 1**). PD1 and Ki67 expression were highest in pT2 patients, followed by pT1 and pTa patients (**Figures 3F, H** and **Supplementary Table 1**). CD8 expression was highest in pT2 patients, and was comparable in pTa and pT1 patients (**Figure 3G** and **Supplementary Table 1**).

### Correlation Between Tumor Microenvironment Markers and Survival in UC

**Figure 4:**
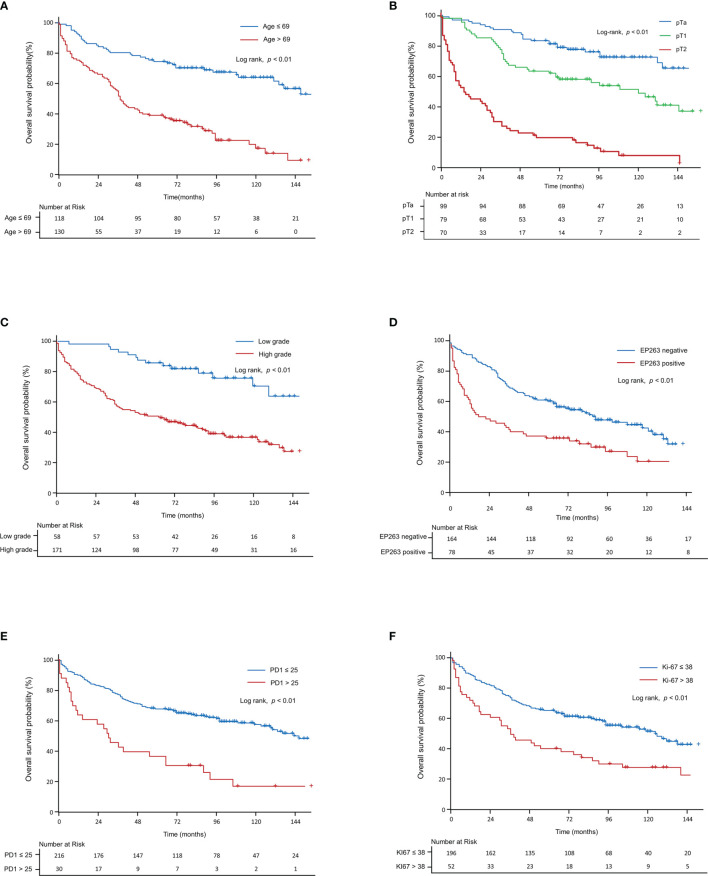
Kaplan–Meier curve for overall survival in all patients. The Kaplan–Meier curves show overall survival according to **(A)** age, **(B)** T-stage, **(C)** grade, **(D)** EP263, **(E)** PD1, and **(F)** Ki67. PD1, programmed cell death protein 1.

**Table 2** shows the results of the univariate and multivariate analyses of OS, including the clinicopathological factors and tumor microenvironment markers in all patients (pTa, pT1, and pT2 stages). Based on the univariate analysis, age (p<0.01), T-stage (p<0.01), tumor grade (p<0.01), EP263 (p<0.01), HER2 (p<0.01), PD1 (p<0.01), CD8 (p<0.05), and Ki67 (p<0.01) were significant prognostic factors. Through the multivariate analysis, we found that age (p<0.01), T-stage (p<0.01), EP263 (p<0.05), PD1 (p<0.01), CD8 (p<0.05), and Ki67 (p<0.05) were significant prognostic factors. **Figure 4** was generated using the Kaplan–Meier survival curves based on significant factors derived from the univariate and multivariate analysis, including age (p<0.01, **Figure 4A**), T-stage (p<0.01, **Figure 4B**), grade (p<0.01, **Figure 4C**), EP263 (p<0.01, **Figure 4D**), PD1 (p<0.01, **Figure 4E**), and Ki67 (p<0.01, **Figure 4F**).

**Table 2:** Univariate and multivariate analyses of OS in all patients.

	Univariate (OS)			Multivariate (OS)		
	HR (95% CI)		p-value	HR (95% CI)		p-value
Age (continuous)	1.070 (1.050–1.090)		<0.01	1.064 (1.043–1.084)		<0.01
Sex	0.950 (0.600–1.490)		0.82			
Bladder tumor						
- T-stage						
Ta	Reference			3.270 (2.360–4.531)		<0.01
T1	2.168 (1.333–3.526)		<0.01			
T2	8.190 (5.159–13.001)		<0.01			
- Grade (High vs. Low)	3.120 (1.820–5.370)		<0.01			
- Concurrent CIS	1.150 (0.560–2.350)		0.71			
Microenvironment markers						
- EP263 (positive vs. negative)	1.970 (1.380–2.800)		<0.01	1.568 (1.025–2.398)		<0.05
- IM263 (positive vs. negative)	1.390 (0.970–1.990)		0.07			
- EP142 (positive vs. negative)	0.940 (0.660–1.330)		0.71			
- IM142 (positive vs. negative)	1.350 (0.950–1.910)		0.09			
- HER2 (positive vs. negative)	1.690 (1.200–2.400)		<0.01			
- PD1 (continuous)	1.020 (1.010–1.030)		<0.01	1.023 (1.007–1.040)		<0.01
- CD8 (continuous)	1.010 (1.000–1.010)		<0.05	0.989 (0.981–0.997)		<0.05
- Ki67 (continuous)	1.010 (1.010–1.020)		<0.01	0.985 (0.974–0.997)		<0.05

OS, overall survival; NMIBC, non-muscle-invasive bladder cancer; HR, hazard ratio; CI, confidence interval; CIS, carcinoma in situ; HER2, human epidermal growth factor receptor-2; PD1, programmed cell death protein 1.

### Correlation of Tumor Microenvironment Markers With RFS and PFS in NMIBC

**Figure 5:**
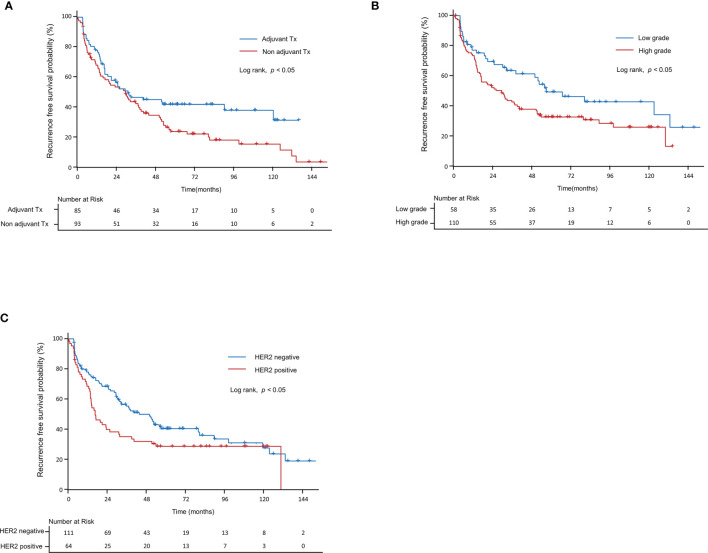
Kaplan–Meier curve for recurrence-free survival in non-muscle-invasive bladder cancer patients. Recurrence-free survival according to **(A)** grade, **(B)** adjuvant therapy, and **(C)** HER2+. HER2, human epidermal growth factor receptor-2.

**Table 3** summarizes the results of the univariate and multivariate analyses for RFS and PFS, including the clinicopathologic factors and tumor microenvironment markers in NMIBC patients. In the univariate analysis, adjuvant therapy (p=0.05), tumor grade (p<0.05), and HER2+ (p<0.05) were evaluated as significant biomarkers for RFS. In multivariate analyses, we found that RFS had a significant correlation with two Cox proportional hazard models: model^a^ and model^b^. Model^a^ was built using adjuvant therapy (p<0.01) and tumor grade (p<0.01), and model^b^ was built using adjuvant therapy (p<0.05) and HER2+ (p<0.05). Using a Kaplan–Meier model, **Figure 5** was generated from significant factors of adjuvant therapy (p<0.05, **Figure 5A**), tumor grade (p<0.05, **Figure 5B**), and HER2+ (p<0.05, **Figure 5C**).

**Table 3:** Univariate and multivariate analyses of RFS and PFS in NMIBC patients.

	Univariate (RFS)		Multivariate (RFS)			Univariate (PFS)		Multivariate (PFS)	
	HR (95% CI)	p-value	HR (95% CI)		p-value	HR (95% CI)	p-value	HR (95% CI)	p-value
Age (continuous)	1.020 (1.000–1.030)	0.07				1.071 (1.023-1.121)	<0.05	1.051 (0.999–1.106) 1.076 (1.024–1.131)	0.05^c^ <0.01^d^
Sex	0.720 (0.420–1.220)	0.22				0.950 (0.280–3.300)	0.94		
Adjuvant therapy	1.454 (0.997–2.120)	0.05	0.582 (0.390–0.870) 1.510 (1.033–2.207)		<0.01^a^ <0.05^b^	0.660 (0.260–1.700)	0.39		
Bladder tumor									
- T-stage	1.300 (0.900–1.880)	0.16				27.73 (3.671–209.4)	<0.01	11.70 (2.660–51.45)	<0.01^c^
- Grade (High vs. Low)	1.605 (1.048–2.458)	<0.05	1.890 (1.215–2.940)		<0.01^a^	10.09 (1.337–76.09)	<0.05		
- Concurrent CIS	1.070 (0.520–2.200)	0.85				1.170 (0.160–8.870)	0.88		
- Tumor size	1.080 (0.730–1.620)	0.69				1.030 (0.380–2.790)	0.95		
- Multiple tumor	1.310 (0.820–2.080)	0.26				0.480 (0.110–2.120)	0.34		
- Solid or Mixed	0.880 (0.470–1.630)	0.68				1.140 (0.260–4.980)	0.86		
Microenvironment markers									
- EP263 (positive vs. negative)	1.300 (0.850–2.000)	0.23				1.360 (0.440–4.170)	0.59		
- IM263 (positive vs. negative)	1.080 (0.740–1.570)	0.70				3.831 (1.244–11.780)	<0.05	3.659 (1.183–11.32)	<0.05^d^
- EP142 (positive vs. negative)	0.790 (0.540–1.160)	0.23				0.890 (0.340–2.290)	0.80		
- IM142 (positive vs. negative)	1.070 (0.730–1.570)	0.73				1.320 (0.520–3.370)	0.56		
- HER2 (positive vs. negative)	1.480 (1.010–2.160)	<0.05	1.532 (1.048–2.238)		<0.05^b^	2.552 (1.006–6.473)	<0.05		
- PD1 (continuous)	1.000 (0.980–1.020)	0.75				0.980 (0.920–1.040)	0.55		
- CD8 (continuous)	1.000 (0.990–1.010)	0.59				1.000 (0.980–1.020)	0.99		
- Ki67 (continuous)	1.083 (0.744–1.576)	0.68				1.002 (0.973–1.032)	0.87		

In the multivariate analysis for RFS, Cox proportional hazard model^a^ included adjuvant therapy and grade, while model^b^ included adjuvant therapy and HER2+.

In the multivariate analysis for PFS, Cox proportional hazard model^c^ included age and grade, while model^d^ included age and IM263.

RFS, recurrence-free survival; PFS, progression-free survival; NMIBC, non-muscle-invasive bladder cancer; HR, hazard ratio; CI, confidence interval; CIS, carcinoma in situ; HER2, human epidermal growth factor receptor-2; PD1, programmed cell death protein 1.

**Figure 6:**
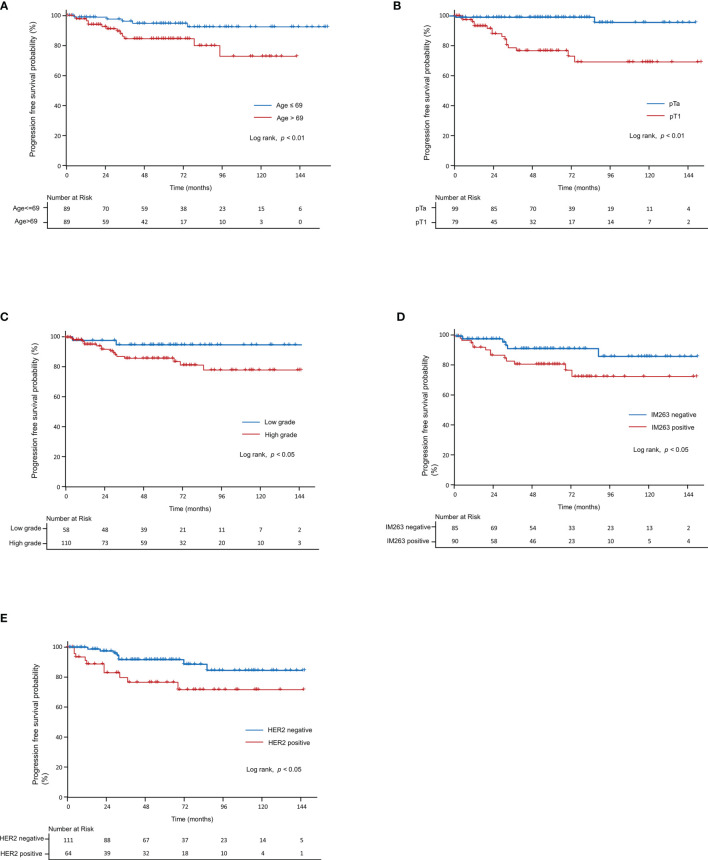
Kaplan–Meier curve for progression-free survival in non-muscle-invasive bladder cancer patients. Progression-free survival according to **(A)** age, **(B)** T-stage, **(C)** grade, **(D)** IM263, and **(E)** HER2+. HER2, human epidermal growth factor receptor-2.

In the univariate analysis of PFS, age (p<0.05), T-stage (p<0.01), tumor grade (p<0.05), and IM263 (p<0.05) were significant prognostic factors. In the multivariate analysis, we found that PFS was significantly correlated with two Cox proportional hazard models: model^c^ and model^d^. Model^c^ was built using age (p=0.05) and T-stage (p<0.01), and model^d^ was built using age (p<0.01) and IM263 (p<0.05). **Figure 6** was generated based on significant factors from the log-rank test of the Kaplan–Meier model that were screened for factors in the univariate analysis, including age (p<0.01, **Figure 6A**), T-stage (p<0.01, **Figure 6B**), tumor grade (p<0.05, **Figure 6C**), IM263 (p<0.05, **Figure 6D**), and HER2+ (p<0.05, **Figure 6E**).

Statistical analyses were performed between HER2 positive and HER2 negative patient groups in the gray area of **Supplementary Figure 1**, based on the tendency of the recurrence-free survival curve appearing to have narrowing trends. Baseline patient characteristics of the HER2 positive group without relapse by 72 months are shown in **Supplementary Table 2**; characteristics of biomarker expression in this subgroup are shown in **Supplementary Table 3**. The proportion of HER2 2^+^ or HER2 3^+^ was significantly higher in patients with relapse or censor before 72 months (p<0.05, **Supplementary Table 3**).

## Discussion

In this study, an increase in bladder cancer stage (Ta–T2) correlated with increased Ki67, PD1, EP263, and IM142 expression levels. The T1 and T2 stages had higher expression levels of HER2 and IM263 than the Ta stage, and there was no significant difference between the expression levels in the T1 and T2 stages. CD8 levels in the Ta and T1 stages were comparable but were lower than those in the T2 stage. No significant differences were noted in the EP142 levels across the three stages. Based on a multivariate analysis model including adjuvant therapy and tumor grade in NMIBC, RFS was associated with HER2+, while OS was associated with age, T-stage, EP263, PD1, CD8, and Ki67 levels.

In NMIBC patients, a higher disease stage correlated with a higher expression of PD1 and EP263. EP263 was found to be a predictive factor for progression. On the contrary, cytotoxic lymphocyte infiltration assessed by CD8 levels did not vary significantly in the different stages of NMIBC; therefore, it does not appear to be a prognostic factor. For over 40 years, adjuvant intravesical BCG has been the gold standard treatment for UC, although its mechanism of action, including the target antigen, remains unclear. However, with the development of PD-L1 therapeutics, there has been an increased interest in this mechanism, and strategies targeting PD-L1 have been approved by the FDA for advanced UC patients (9, 10, 21). In a study on NMIBC, patients with advanced disease showed higher levels of PD-L1 in BCG granulomas. Although there is no conclusive evidence that T-cell-related immunity is related to BCG treatment, this may hint at a link between BCG failure and T-cell depletion (22). Therefore, the systemic immune-related adverse effects of ICBs should be considered when treating BCG-resistant UC. An evaluation of the levels of tumor microenvironment markers at baseline can help predict the outcome of ICB treatment in NMIBC patients. While the relationship between PD1 and CD8 has been widely reported for MIBC (23, 24), only a few reports describe their association in NMIBC (25, 26). Therefore, while EP263 has the potential to be a prognostic marker for progression, CD8 appears to have a limited role in these patients.

The reason for conflicting results with respect to measurement results of the TME in bladder cancer was that, due to molecular heterogeneity, different TMEs were reported to coexist, even in the case of bladder cancer extracted from a single mass (27). Furthermore, due to co-localization of PD-L1, PD-1, and CD8+ cell characteristics, other reasons may include the presence of parts rich or low in PD-L1, depending on the part measured by the TME environment (28, 29). In addition to these tumor-related biases that were partly extracted, problems due to test methods and scoring were present; however, there is no standardization of tests for PD-L1 IHC expression, and there were several analyses utilizing different clones and scoring algorithms (30). Currently, four commercially available antibodies are SP142 (Ventana), SP256 (Ventana), 22C3 (Dako), and 29-8 (Dako). In this study, we used a Ventana analysis system of PD-L1 antibodies (SP142, SP256) system. SP142 antibody is the most widely used method of PD-L1 because the immune cell (IC) count score measured from SP142 is indicates use of atezolizumab (Tecentriq^®^) in muscle invasive bladder cancer; furthermore, since SP142 is specifically designed to stain IC, it is advantageous in being more sensitive and allowing for a clearer view of immune infiltrates (31). In a previous study using the above four antibodies in muscle invasive specimens, a concordance rate of 80-90% was reported; however, some specimens that used IM142 antibody reported inconsistency in the staining pattern that varied from other specimens (32). and Some reports criticized SP142 for poor reproducibility compared to other PD-L1 antibodies (33) The authors designed the study to use multiple antibodies from the same tumor rather than relying on a single slide to reduce bias in the testing methods for these antibodies. In NMIBC particularly, each marker was analyzed for each prognosis competitively because there was no report on concordance or prognostic significance according to use of each antibody in NMIBC.

We evaluated the role of high HER2 expression in the recurrence of superficial UC. HER2, a mediator of tumor cell growth through the regulation of a tyrosine kinase, is amplified in UC, as shown by genome sequencing (34) and immunohistochemistry (35). HER2 overexpression is a biomarker that correlates with poor prognosis and represents a potential target marker for anti-HER2 therapy based on experience with breast cancer or gastric cancer (36, 37). In order for HER2-related therapeutics to be effective in bladder cancer, investigation of HER2 expression is needed because HER2 expression is directly related to the treatment mechanism. The relationship between HER2 expression and bladder cancer that is known to date has been reported at the level that correlated with advanced bladder cancer. It was reported that HER2 overexpression was already observed in a specimen before progression, and was consequently observed after progression (38, 39); genetic changes also showed similar patterns to these reports (40). Therefore, the known onset point of HER2 expression was at the level of previous advanced bladder cancer lesions. This study revealed HER2 overexpression is also observed at a level of early diagnosis of NMIBC and correlates with bladder cancer recurrence and progression. The recent emergence of novel ADCs has renewed the interest in HER2-targeted therapies for UC. Since ADCs deliver high concentrations of a cytotoxic agent to tumor cells with specific target antigens, they effectively kill tumor cells with minimal systemic side effects. Most agents targeting HER2 require genome amplification of HER2 for effective treatment (34). However, studies on RC48-ADC in IHC 2^+^ or IHC 3^+^ UC patients suggest that only a moderate level of protein expression is sufficient to induce a response (15). In line with these findings, this study provides information on HER2 expression as a prognostic biomarker in NMIBC, thus identifying high-risk patients with this biomarker and providing a potential patient group that may be targeted for anti-Her2 therapies. In particular, among HER2 2^+^ or HER2 3^+^ characteristics; only two patients without recurrence or a 72-month censor were followed-up. Hence, we recommend intensive surveillance for patients with initially expressed HER2 2^+^ or HER2 3^+^. Further research is needed to confirm these results and to explore whether HER2 is an effective target antigen of this treatment.

Oportuzumab monatox (OM), an ADC that uses Pseudomonas exotoxin A (ETA 252-608) as a cytotoxic agent and targets EpCAM expressing tumor cells, has shown potential as an intravesical therapeutic agent (16). Phase II trials of OM for BCG-refractory NMIBC have demonstrated its potential as a method of intravesical therapy (16). Unlike mUC and MIBC, NMIBC has a relatively good prognosis, with a few exceptions (2). Studies on the natural course of NMIBC patients have evaluated various clinicopathological factors that can predict recurrence and progression. Although a guideline has been developed for appropriate adjuvant therapy (2), many NMIBC patients are at risk of repeated relapses and progression (4, 5). Furthermore, due to the current chronic shortage of BCG, urology oncologists often have to treat patients not based on these guidelines (2) but on the amounts of BCG available at their hospitals (41). Under these circumstances, ADCs are promising intravesical drugs that directly exert cytotoxic effects on tumor cells by targeting specific tumor antigens, with reduced systemic adverse effects (16). ADCs are particularly beneficial for patients whose cancer risk is not high enough to tolerate the systemic side effects or those who developed side effects when treated with BCG. In this study, HER2+ expression showed a significant association with RFS and is therefore a potential target antigen for the development of ADC-mediated intravesical therapeutic agents and an alternative adjuvant therapy to BCG.

Ki67 is a DNA-bound protein activated only during the proliferation phase and is not detected during cell cycle quiescence. Studies on Ki67 in UC have reported it as a prognostic factor for recurrence and progression in upper tract UC (19, 42) NMIBC after TURB (43, 44). In the present study, we found a correlation between higher stages of UC and increased Ki67 expression levels. Ki67 was analyzed as a significant prognostic factor in a survival model including patients with all stages of UC but was not a prognostic factor for recurrence or progression in NMIBC patients. Patients who undergo radical surgery, such as the upper ureter tract UC, can be classified into risk groups based on the pathology of the full ureter’s layers, which is a better indicator of the depth of tumor invasion than the TURB specimen. In this patient group, most cases of recurrence occur in the form of local recurrence, and since most cases of local recurrence are explained by pathological results of surgically removed ureter cancer, the pathologic results have a close relationship with patient prognosis (19, 42). However, in NMIBC patients, many recurrences are not related to primary UC that was surgically removed, such as by incomplete TURB. For the reasons mentioned here, unlike previous studies reporting that Ki67 is related to prognosis under certain conditions, in NMIBC (43, 44), we found no significant relationship between Ki67 expression levels and recurrence/progression.

A limitation of this study is that the number of patients included was relatively small; therefore, it will be necessary to assess whether the results can be replicated in a large-scale study. In addition, since this was a retrospective study, the stored samples had been stained and previously handled. All formalin-fixed and paraffin-embedded specimens were archived in closed wooden boxes, protected from light, in a dry dedicated deposit at constant room temperature between 18-25°C. In our study, as at 2020, for immunostaining, there were two specimens stored for 20 years. Including the two cases, 32 cases in total had been archived for more than 15 years. We performed immunostaining for Vimentin, which is known to efficiently reflect preserved immunoreactivity of the old paraffin block specimen for all the cases included in the study, and confirm whether mesenchymal cells and endothelial cells were well stained. Moreover, in order to obtain good immunostaining properties, at least 500 micrometers were cut out during the TMA block manufacturing process and immunostaining was performed on the deep portion of the tissue. Two Vimentin-stained specimens were stored for 20 years (**Supplementary Figure 2**). Therefore, although only the results from well-preserved samples were included in the study, errors caused by the use of old samples could not be avoided. There are also limitations in the analysis due to the assumption of a causal relationship between expression level and prognosis. However, although performed on a small-scale, this was a well-designed study in the field of therapeutic agent development, which has received increasing interest in recent years. Several studies have reported that PD-L1 or HER2 expression varies depending on the patient’s status, such as additional treatment for tumors and site of lesion collection (primary or metastatic) (45–47). The diagnostic criteria for “high PD-L1 expression” depends on the staining method, antibodies used, and infiltrating cells (tumor or immune cells) (48). PD-L1 expression has been shown to increase after chemotherapy in patients with mUC (46). A study on patients with recurrent NMIBC reported increased HER2 expression when cancer relapsed after adjuvant intravesical therapy (45). Moreover, since metastatic lesions have higher HER2 expression than primary lesions (47), results might vary depending on adjuvant therapy or status of recurrence. In this study, we included only patients diagnosed with UC for the first time with no history of UC-related diagnosis or therapy. Therefore, our results are significant because they reveal the initial changes in the tumor microenvironment at different stages of the disease. We also evaluated the prognostic role of these tumor microenvironment markers by comparing them with other clinicopathological factors to identify their potential as therapeutic agents. Given the shortage of BCG, we believe our findings will help identify a target antigen for developing therapies alternative to BCG for the treatment of NMIBC.

## Conclusions

In NMIBC patients, the expression of IM263, an immune cell marker, and HER2 increased as the disease stage increased. IM263 and HER2 levels were significant predictors of PFS and RFS, respectively, suggesting their potential as biomarkers for progression and recurrence. Additionally, HER2 is a promising target antigen for use in an ADC for adjuvant intravesical therapeutics as an alternative to BCG.

## Data Availability Statement

The original contributions presented in the study are included in the article/**Supplementary Material**. Further inquiries can be directed to the corresponding authors.

## Ethics Statement

The studies involving human participants were reviewed and approved by Institutional Review Board of Gangneung Asan Hospital. The ethics committee waived the requirement of written informed consent for participation.

## Author Contributions

SJK and D-WE were responsible for conceptualization and design. Acquisition of data was conducted by SL, B-JN, and GK. HC, SJK, and D-WE analyzed and interpreted the data. HC and SJK drafted the manuscript. WN, HK, SWK, JP, SJK, and D-WE were responsible for critical revision of the manuscript. SJK and D-WE supervised the project. All authors contributed to the to the article and approved the submitted version.

## Funding

This work was supported by the Gangneung Asan Hospital Medical Institute and the Asan Foundation under Grant number 2020–IB008.

## Conflict of Interest

The authors declare that the research was conducted in the absence of any commercial or financial relationships that could be construed as a potential conflict of interest.

## Publisher’s Note

All claims expressed in this article are solely those of the authors and do not necessarily represent those of their affiliated organizations, or those of the publisher, the editors and the reviewers. Any product that may be evaluated in this article, or claim that may be made by its manufacturer, is not guaranteed or endorsed by the publisher.
