# Supplementary material for: Identification of New Prognostic Markers and Therapeutic Targets for Non-Muscle Invasive Bladder Cancer: HER2 as a Potential Target Antigen
Source: Front Immunol. 2022 May 23;13:903297. doi: 10.3389/fimmu.2022.903297 (PMC9167936; doi:10.3389/fimmu.2022.903297)

Supplementary Material

# Supplementary Table

Supplementary Table 1. Baseline expression of tumor microenvironment markers according to T-stage

|  | Total | pTa | pT1 | pT2 | p-value |
| --- | --- | --- | --- | --- | --- |
| EP263 positive | 78 (32.2%) | 11 (11.2%) | 29 (38.2%) | 38 (55.9%) | <0.01^*^ |
| IM263 positive | 142 (58.4%) | 39 (40.2%) | 51 (65.4%) | 52 (76.5%) | <0.01^*^ |
| EP142 positive | 104 (42.4%) | 37 (37.8%) | 36 (46.8%) | 31 (44.3%) | 0.46^*^ |
| IM142 positive | 122 (50.6%) | 30 (31.9%) | 41 (53.2%) | 51 (72.9%) | <0.01^*^ |
| HER2 positive | 103 (42.4%) | 24 (24.7%) | 40 (51.3%) | 39 (57.4%) | <0.01^*^ |
| PD1, *n* | 10.6±18.2 | 4.0±10.3 | 8.4±9.9 | 22.3±26.7 | <0.01^**^ |
| CD8, *n* | 32.9±35.9 | 22.7±25.7 | 29.6±25.9 | 50.9±49.3 | <0.01^**^ |
| Ki67 (%) | 22.1±19.2 | 11.6±11.9 | 24.2±18.3 | 34.5±20.6 | <0.01^**^ |

^*^Pearson's χ^2^ test or Fisher's exact test; ^**^Kruskal–Wallis test

HER2, human epidermal growth factor receptor-2; PD1, programmed cell death protein 1

# Supplementary Table

Supplementary Table 2. Baseline patient characteristics according to the observation time of the 72-month cutoff for HER2 positive non-muscle invasive bladder cancer

|  | Total | Under 72 | Over 72 | p-value |
| --- | --- | --- | --- | --- |
| Number of patients, n (%) | 64 (100) | 51 | 13 |  |
| Age, years (mean±SD) | 70.6±10.7 | 71.3±10.5 | 67.8±11.3 | 0.29 |
| Sex, n (%), Male | 54 (84.4%) | 45 (88.2%) | 9 (69.2%) | 0.19 |
| pT1 stage, n (%) | 40 (62.5) | 34 (66.7) | 6 (46.2) | 0.21 |
| Tumor description, n (%) |  |  |  | 0.50 |
| Papillary | 55 (85.9%) | 44 (86.3%) | 11 (84.6%) |  |
| Solid | 3 (4.7%) | 3 (5.9%) | 0 (0.0%) |  |
| Mixed | 6 (9.4%) | 4 (7.8%) | 2 (15.4%) |  |
| Tumor grade, n (%) |  |  |  | 0.38 |
| Low | 9 (14.5) | 6 (12.2) | 3 (23.1) |  |
| High | 53 (85.5) | 43 (87.8) | 10 (76.9) |  |
| Not reported | 2 | 2 | 0 (0.0%) |  |
| Tumor number, n (%) |  |  |  | 0.91 |
| 1 | 34 (64.2) | 28 (66.7) | 6 (54.5) |  |
| 2–4 | 18 (34.0) | 13 (31.0) | 5 (45.5) |  |
| >4 | 1 (1.9) | 1 (2.3) | 0 (0.0) |  |
| Not reported | 11 | 9 | 2 |  |
| Tumor size (cm), n (%) |  |  |  | 0.53 |
| 1 | 7 (12.7) | 5 (11.4) | 2 (18.2) |  |
| 1–3 | 23 (41.8) | 21 (47.7) | 2 (18.2) |  |
| >3 | 25 (45.5) | 18 (40.9) | 7 (63.6) |  |
| Not reported | 9 | 7 | 2 |  |
| Concurrent CIS, n (%) | 58 (90.6) | 46 (90.2) | 12 (92.3) | 1.00 |
| Adjuvant therapy, n (%) |  |  |  | 0.66 |
| BCG | 30 (46.9) | 23 (45.1) | 7 (53.8) |  |
| Mitomycin | 3 (4.7) | 2 (3.9) | 1 (7.7) |  |
| None | 31 (48.4) | 26 (51.0) | 5 (38.5) |  |
| Recurrence, n (%) | 46 (71.9) | 45 (88.2) | 1 (7.7) | <0.01 |
| Progression, n (%) | 10 (15.6) | 10 (19.6) | 0 (0.0) | 0.19 |
| Expire, n (%) | 30 (46.9) | 28 (54.9) | 2 (15.4) | <0.05 |

^*^Analysis of variance; ^**^Kruskal–Wallis; ^***^ Pearson's χ-2 test or Fisher's exact test

Abbreviations: SD, standard deviation; BMI, body mass index; DM, diabetes mellitus; HTN, hypertension; CIS, carcinoma in situ; BCG, Bacillus Calmette–Guérin

# Supplementary Table

Supplementary Table 3. Baseline expression of tumor microenvironment markers according to the observation time of the 72-month cutoff for HER2 positive non-muscle invasive bladder cancer

|  | Total (n=64) | Under 72 (n=51) | Over 72 (n=13) | p-value |
| --- | --- | --- | --- | --- |
| EP263 positive | 21 (32.8%) | 18 (35.3%) | 3 (23.1%) | 0.61^*^ |
| IM263 positive | 39 (60.9%) | 32 (62.7%) | 7 (53.8%) | 0.52 |
| EP142 positive | 26 (40.6%) | 21 (41.2%) | 5 (38.5%) | 1.00^*^ |
| IM142 positive | 29 (45.3%) | 24 (47.1%) | 5 (38.5%) | 0.61^*^ |
| HER2 |  |  |  | <0.05^***^ |
| HER2 1^+^ | 37 (57.8%) | 26 (51.0%) | 11 (84.6%) |  |
| HER2 2^+^ | 17 (26.6%) | 16 (31.4%) | 1 (7.7%) |  |
| HER2 3^+^ | 10 (15.6%) | 9 (17.6%) | 1 (7.7%) |  |
| PD1, n | 7.4±10.6 | 7.4±11.4 | 7.6±7.4 | 0.94 |
| CD8, n | 27.5±25.7 | 27.5±26.1 | 27.7±25.0 | 0.98 |
| Ki67 (%) | 25.6±17.5 | 26.0±18.2 | 24.2±15.2 | 0.75 |

^*^Pearson's χ^2^ test or Fisher's exact test; ^**^Kruskal–Wallis test; ^***^ Fisher's exact test (HER2 1^+^ vs. HER2 2^+^, 3^+^)

HER2, human epidermal growth factor receptor-2; PD1, programmed cell death protein 1

# Supplementary Figure

Supplementary Figure 1. Kaplan–Meier curve for recurrence-free survival in non-muscle invasive bladder cancer patients according to HER2 +

Abbreviations: HER2, human epidermal growth factor receptor-2


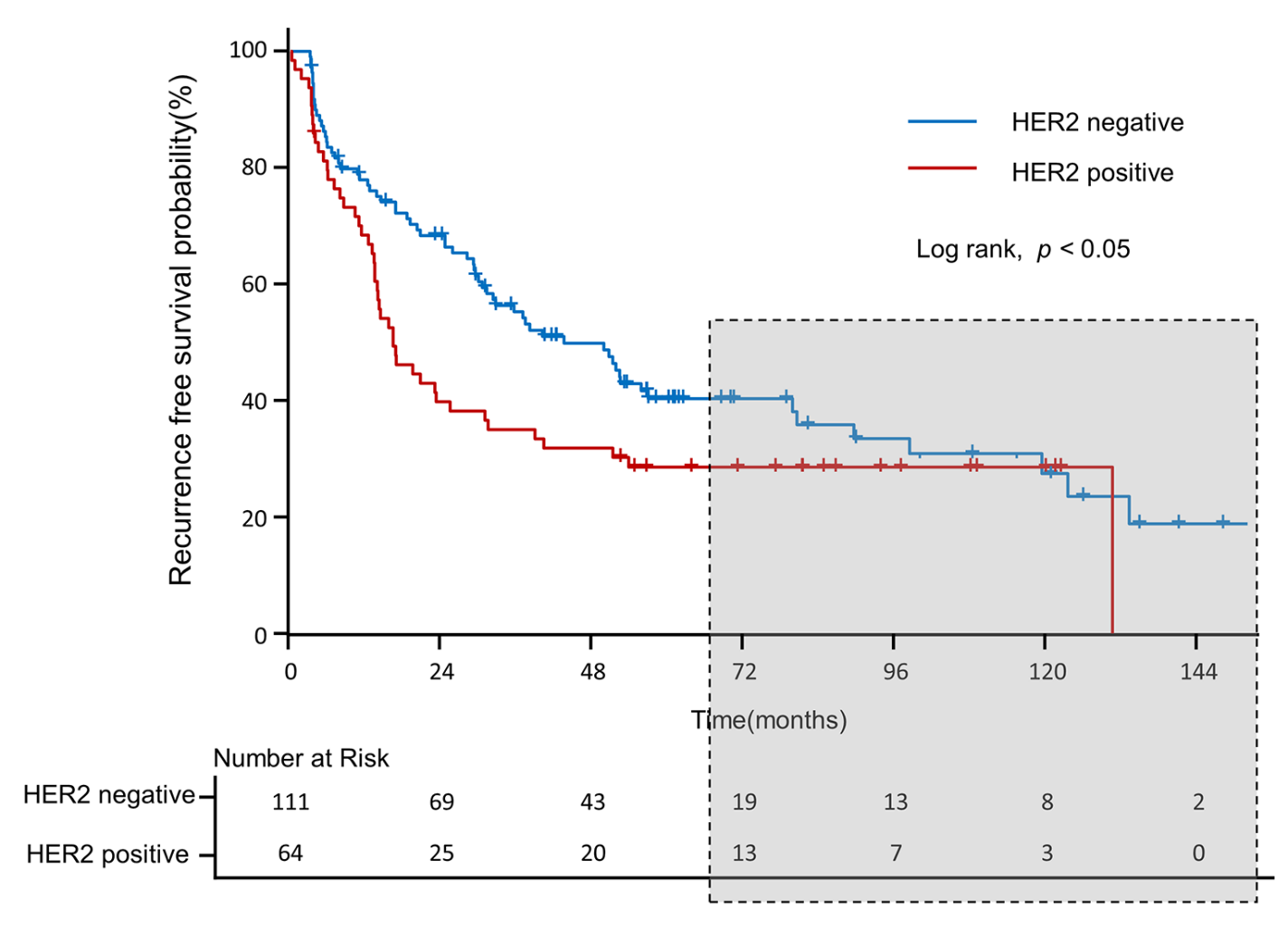


# Supplementary Figure

Supplementary Figure 2. Vimentin stains for two specimens stored for 20 years


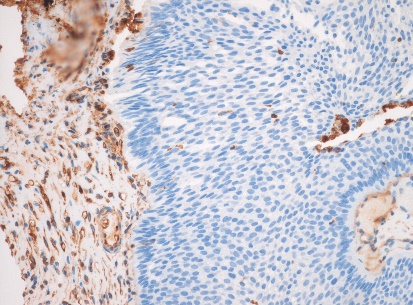

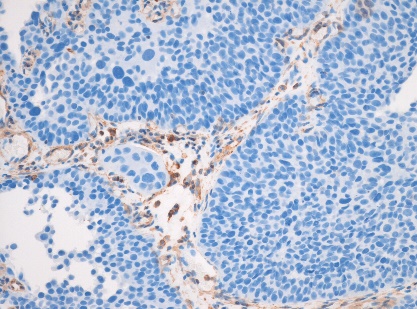

Supplement: Supplementary file 1 [file DataSheet_1.docx]
